# The role of insulin in transdifferentiated hepatocyte proliferation and function in serum‐free medium

**DOI:** 10.1111/jcmm.14303

**Published:** 2019-04-04

**Authors:** Ce Gu, Panpan Li, Wei Liu, Yan Zhou, Wen‐Song Tan

**Affiliations:** ^1^ State Key Laboratory of Bioreactor Engineering East China University of Science and Technology Shanghai P. R. China

**Keywords:** BAL, hepatocyte function, insulin, proliferation, serum‐free medium, transdifferentiated hiHeps

## Abstract

Transdifferentiated hepatocytes are potential seeding cells for bioartificial liver (BAL) treatment, and it is important to obtain a sufficient number of functional hepatocytes in serum‐free medium (SFM). Although insulin plays an essential role in promoting cell proliferation and metabolism, the functions of insulin in transdifferentiated cells remain poorly understood. Here, we found that 1.0 mg/L insulin significantly increased human‐induced hepatocyte‐like cells (hiHeps) proliferation and viability in SFM. The pro‐proliferative effect of insulin on these cells occurred via augmented cyclin D1 expression that was mediated by activation of the Akt1/mTOR/p70S6K and Akt1/P53 pathways. Further studies revealed that insulin also enhanced the specific liver function of hiHeps in SFM. Additionally, Western blotting and siHNF1A transfection analysis showed that insulin increased the protein expression of Albumin (ALB) and UDP‐glucuronosyltransferase1A1 （UGT1A1 ） in hiHeps via *HNF1A*. Finally, hiHep proliferation and the expression of specific genes were maintained during long‐term passaging in SFM supplemented with 1.0 mg/L insulin. Collectively, our findings show that insulin promotes transdifferentiated hiHep proliferation and specific functional expression. These findings have important implications for the expansion of functional hiHeps prior to clinical applications of BALs.

## 1 INTRODUCTION

Acute liver failure (ALF) is a life‐threatening disease caused by hepatocellular dysfunction and has high‐mortality rates.^1^ Currently, liver transplantation is the most effective treatment strategy for ALF. However, because of the limited availability of suitable donor organs, bioartificial livers (BALs) have attracted much attention and have become a promising therapy for the treatment of ALF.^2^ BALs are mainly composed of functional hepatocytes, bioreactors and extracorporeal circulation devices, and the core of the treatment is functional hepatocytes.^3^ Therefore, it is particularly important to obtain a sufficient number of functional hepatocytes for future BAL applications.

Human‐induced hepatocyte‐like cells (hiHeps) are transdifferentiated from human skin fibroblasts by lentiviral expression of FOXA3, HNF1A, HNF4A and SV40 large T, which share numerous characteristics with mature hepatocytes, such as UGT enzymatic activity, cytochrome P450 enzymatic activity and synthesis of glycogen and albumin. In addition, hiHeps were demonstrated to display a typical epithelioid phenotype and to be capable of expansion in vitro.^4^ As the advantages of the abundant sources and low rates of immunological rejection, hiHeps represent an important in vitro cell source for BAL treatment. Previous studies have developed a hiHep‐based BAL system (hiHep‐BAL) for the treatment of porcine ALF, which indicated that BAL could be applied to clinical treatment because it attenuated liver damage, restored porcine liver function and prolonged survival.^5^ However, for hiHeps to be used with a BAL for clinical treatment, several criteria need to be met including the generation of at least 10^10^ functional hiHeps. Additionally, the expansion of these cells must be performed in a chemically defined SFM in vitro prior to clinical applications.^2^, ^6^, ^7^ Currently, the culture and expansion of hiHeps are performed in hepatocyte maintenance medium (HMM) containing fetal bovine serum (FBS) in vitro. FBS has a considerable degree of inter‐batch variation and may cause infection and immunological responses following cell treatment.^7^, ^8^ Therefore, serum‐free strategies using exogenous growth factors have been used to achieve the clinical‐scale production of cells, and these growth factors can be applied to regulate cell proliferation and function.^9^, ^10^

Previously, we preliminarily screened a chemically defined serum‐free medium (SFM) through Plackett‐Burman design,^11^ and then obtained a medium through initial optimization, named GCM14 medium, but it merely maintained hiHep growth and certain functions. Therefore, it is necessary to add exogenous factors to further optimize the medium. As insulin is a peptide hormone having several different actions, not only on glucose metabolism, but also on regulating cell proliferation.^12^, ^13^, ^14^ For example, in the liver, insulin can promote glycogen synthesis, lipid synthesis and protein production while turning off gluconeogenesis.^15^, ^16^ In primary rat hepatocytes, a study showed that insulin is an important regulator of liver‐specific cytochrome P450 gene expression, and the level of albumin mRNA expression was reduced when insulin was lacking.^17^ In regard to regulating cell proliferation, a study revealed that insulin‐stimulated goose liver cell mRNA and protein expression of genes involved in proliferation and promoted cell growth through the PI3K/AKT signalling pathway.^14^, ^18^ Similarly, insulin can induce CARM1 up‐regulation to facilitate rat primary hepatocyte proliferation and enhance cyclin‐dependent kinase expression.^19^ Although the results in the literature were sometimes inconsistent,^20^, ^21^ in most instances, insulin was shown to promote the proliferation of different cell types.^22^, ^23^, ^24^, ^25^ Thus, it has been demonstrated that insulin plays a crucial role in regulating cell proliferation and metabolism. However, the effect and mechanism of insulin on the proliferation and function of human hepatocytes, especially transdifferentiated cells, remain poorly understood.

In this study, the effects of insulin on the proliferation and function of hiHeps cultured in a defined SFM were investigated. We found that 1.0 mg/L insulin significantly promoted hiHep proliferation and maintained cell viability, and this pro‐proliferation effect occurred via augmented cyclin D1 expression that was mediated by the Akt1/mTOR/p70S6K and Akt1/p53 pathways. Additionally, insulin stimulated the hiHep albumin synthesis and secretion, lipid synthesis, glucose uptake and glycogen synthesis in SFM. Finally, we found that insulin increased the protein expression of Albumin (ALB) and UDP‐glucuronosyltransferase1A1（UGT1A1） in hiHeps by affecting the level of *HNF1A*. These results provide a better understanding of how insulin affects transdifferentiated hiHeps, which has important implications for utilizing insulin to expand clinical‐grade, functional hiHeps in vitro for use in BALs.

## 2 MATERIALS AND METHODS

### 2.1 Cell source and culture medium

hiHeps were obtained from the Shanghai Institutes for Biological Sciences of the Chinese Academy of Sciences. hiHeps were cultured in HMM, which consisted of DMEM/F12 (Gibco, USA) basal culture medium supplemented with 1% (v/v) FBS (Hyclone, USA), 40 ng/mL transforming growth factor (TGF‐α, PeproTech, NJ, USA, 40 ng/mL epidermal growth factor (EGF, PeproTech, USA), 10 µmol/L dexamethasone (DEX, Sigma‐Aldrich, USA), 1% (v/v) 1 × ITS (Gibco, USA), 0.1 mg/mL ornithine (Sigma‐Aldrich, USA), 0.03 mg/mL proline (Sigma‐Aldrich), and 0.61 mg/mL nicotinamide (Sigma‐Aldrich, USA).

The designed and initial optimized SFM was named GCM14 medium, which consisted of DMEM/F12 (Gibco, USA) basal culture medium supplemented with 10 ng/mL TGF‐α (PeproTech, USA), 40 ng/mL EGF (PeproTech, USA), 10 µmol/L DEX (Sigma‐Aldrich, USA), 1.0 mg/L α‐Linolenic acid (Solarbio, China), 0.1 mg/L Vitamin A acetate (Solarbio, China) and 1.0 mg/L Vitamin E acetate (Solarbio, China).

### 2.2 Cell proliferation assay

To assess the effect of insulin on cell proliferation, hiHeps were analysed using a cell counting kit‐8 (CCK‐8, DOJINDO, Japan) according to the manufacturer's instructions. Cell proliferation was expressed as a percentage relative to the proliferation of the non‐treatment control, which was set to 100%. For each group, mean values of the absorbance from three wells were calculated.

### 2.3 Flow cytometric analysis

hiHeps were cultured in 6‐well plates at 1 × 10^6^ cells/well in GCM14 medium supplemented with or without 1.0 mg/L insulin. After 4 days, the cells were harvested and centrifuged at 800 rpm and 4°C for 5 minutes, washed with cold PBS twice and fixed in precooled 70% ethanol at 4°C overnight. After fixation, the cells were washed three times with PBS, resuspended in 500 μL PBS containing 1 μL Triton X‐100 for 30 minutes at room temperature, and then treated with 1 mL of the cell cycle staining reagent propidium iodide (PI, Sigma‐Aldrich, USA) for 30 minutes in the dark. Subsequently, the cell cycle distribution was analysed using a flow cytometer, and the percentage of cells in each phase of the cell cycle was determined using FlowJo software. Three independent biological replicates were performed for each experimental group.

### 2.4 Albumin and urea analysis

During cell culture, the supernatant of the medium was collected every two days. The urea concentration in the supernatant was measured using a BUN kit (Nanjing Jiancheng Bioengineering Institute, China), and albumin secreted by hiHeps was measured with a human albumin enzyme‐linked immunosorbent assay kit (Elisa, Bethyl, USA) according to the manufacturer's instructions.

### 2.5 Immunofluorescence staining

Immunohistochemistry was performed as described previously. Briefly, harvested cells were fixed in 4% (w/v) paraformaldehyde for 10 minutes. After fixation, the cells were washed with PBS three times for 5 minutes. Then, the cells were permeabilized with 0.4% (w/v) Triton X‐100 (Invitrogen, USA) for 15 minutes and incubated with 1% BSA at room temperature for 30 minutes to block non‐specific antigens on the cell surface. Then, the cells were incubated with primary antibodies (anti‐human albumin monoclonal antibodies, 1:50 diluted in BSA‐PBS, Bethyl, TX, USA) at 4°C overnight followed by incubation with secondary antibodies (donkey anti‐goat IgG, 1:100 diluted in BSA‐PBS, Neomarker, CA, USA) for 1 hour. Finally, nuclei were stained with DAPI (Invitrogen, USA) for 10 minutes in the dark. Images were taken with a fluorescence confocal microscope.

### 2.6 Detection of glycogen and lipids

Glycogen staining was performed with a Periodic Acid‐Schiff kit (PAS, Sigma‐Aldrich, USA), and the amount of glycogen in hiHeps was quantified using a glycogen content assay kit (Solarbio, BC0340, China) according to the manufacturer's instructions. Similarly, lipid staining was performed with an Oil Red O stain kit (Solarbio, G1262, China).

### 2.7 Uptake of indocyanine green (ICG) and low‐density lipoprotein (LDL)

hiHeps were incubated with 5 mg/mL indocyanine green (ICG, J&K, China) in GCM14, GCM14I and HMM at 37°C for 15 minutes. After washing three times with PBS, images of cellular uptake were captured by microscopy. Similarly, hiHeps were incubated with 30 µg/mL Dil‐ac‐LDL (ANGYUBIO, China) for 5 hours. Then, DAPI (1:100, Invitrogen, USA) was added to the cultures, and the cultures were incubated for 1 hour. Images were taken with a fluorescence microscope.

### 2.8 Measurement of LDH and ALT activity

The lactate dehydrogenase (LDH) and alanine aminotransferase (ALT) levels in the supernatant were determined by an LDH assay kit‐WST (DOJINDO, Japan) and ALT assay kit (Nanjing Jiancheng, China), respectively, according to the manufacturer's instructions.

### 2.9 Analysis of metabolic activity of UGT1A1

To test UDP‐glucuronosyltransferase 1A1 (UGT1A1) activity, hiHeps were incubated with Hank's Balanced Salt Solution (HBSS) containing the indicated drug for 4 hours. The supernatant was collected and measured under a fluorescence microplate reader at wavelengths of 340 and 460 nm.

### 2.10 Quantitative real‐time polymerase chain reaction (qRT‐PCR)

Total RNA was extracted from hiHeps using Trizol reagent (Invitrogen, USA), and reverse transcription of 1 mg RNA was performed with SuperScript III reverse transcriptase (Invitrogen, USA) according to the manufacturer's instructions. The RT‐PCR experiment was performed as follows: 95°C for 10 minutes, and 40 cycles of PCR amplification. The results for each individual reaction were repeated three times and the results were averaged. The primer sequences are shown in Table 1.

**Table 1:** Primers sequences for qRT‐PCR

Gene	Forward (5’‐3’)	Reverse (5'‐3')
HNF1A	CACCAAGCAGGTCTTCACCTC	TCTCGATGACGCTGTGGTTG
FOXA3	AGTGGAGCTACTACCCGGAG	CGATACCCCTTCGGCATCTC
HNF4A	TGCGACTCTCCAAAACCCTC	TCGAGGCACCGTAGTGTTTG
ALB	GCACAATGAAGTGGGTAA	TACTGAGCAAAGGCAATC
AAT	TATGATGAAGCGTTTAGGC	CAGTAATGGACAGTTTGGGT
CK18	CCTACAAGCCCAGATTGCCA	CCAACCTCAGCAGACTGTGT
TF	TGTCTACATAGCGGGCAAGT	GTTCCAGCCAGCGGTTCT
UGT1A1	GCTGGGAAGATACTGTTGATCC	GCCAGACAAAAGCATAGCAGAG
GAPDH	CCACCTTTGACGCTGGG	CATACCAGGAAATGAGCTTGACA

### 2.11 Western Blotting

hiHeps were harvested and lysed with 100 μL protein extraction reagent RIPA lysis buffer (Beyotime, China) containing 10 mmol/L phenylmethylsulfonyl fluoride (PMSF, Beyotime, China) on ice for 30 minutes. The supernatant of each sample was collected by centrifugation at 12,000 rpm for 30 minutes at 4°C, and protein concentrations were quantified using a BCA assay (Beyotime, China), following the manufacturer’s instructions. After being heated for 5 minutes at 95°C in sample buffer (Beyotime, China), protein electrophoresis was performed with 10% SDS‐PAGE gels. Proteins were transferred to PVDF membranes (Millipore) at 100 V for 100 min using a blotting apparatus. Membranes were blocked with 5% (w/v) non‐fat dry milk in TBST buffer (20 mmol/L Tris‐HCl, pH 7.4, 150 mmol/L NaCl, 0.05% Tween‐20) for 30 minutes at room temperature and incubated with primary antibodies (Table 2) overnight at 4°C . Then, the membranes were washed with PBST for 5 minutes for a total of five times and were detected with the appropriate secondary antibodies conjugated with horseradish peroxidase (HRP) (1:10,000, Abcam) at room temperature for 1 hour and were visualized by enhanced chemiluminescence (Millipore). β‐actin served as the internal control, and the results of the Western blotting were analysed by calculating the gray value.

**Table 2:** Antibodies used in Western blot analyses

Antibody/marker	Dilution	Source	Code number
CyclinD1	1:1000	CST	#2978
Phospho‐ERK1/2	1:1000	CST	#4377
ERK1/2	1:1000	CST	#4695
Phospho‐AKT(Ser473)	1:1000	CST	#4060
AKT	1:1000	CST	#4691
Phospho‐p53(Ser15)	1:1000	CST	9284T
p53	1:1000	CST	2527T
Phospho‐mTOR(Ser2448)	1:1000	CST	D9C2
mTOR	1:1000	CST	7C10
Phospho‐p70S6 kinase(Thr389)	1:1000	CST	#9234
p70S6 kinase	1:1000	CST	#2708
UGT1A1 antibody	1:1000	Abcam	ab170858
HNF1A antibody	1:1000	Absin	abs136700
Human serum Albumin MAb	1:1000	R&D system	MAb1455

### 2.12 siRNA transfection and the effect of HNF1A knockdown on ALB and UGT1A1 expression

Cells were transfected with Lipofectamine RNAiMAX according to the manufacturer's protocol (Invitrogen, USA). siRNAs targeting the human *HNF1A* gene (5′‐GCUAGUGGAGGAGUGCAAUTT‐3′, 5′‐AUUGCACUCCUCCACUAGCTT‐3′) (RiboBio, Guangzhou, China) were used to specifically down‐regulate *HNF1A* in hiHeps, and non‐specific sequence siRNAs were used as negative controls (NCs) (RiboBio, China). Cells that did not undergo transfection served as the control, and cells treated with the transfection reagent were considered mock transfected. After 24 to 48 hours of siRNA transfection, the knockdown efficiency was confirmed by qRT‐PCR and Western blot analysis.

### 2.13 Statistical analysis

The results are shown as the mean ± standard deviation (SD), with at least three separate experiments for each study. Statistical significance was evaluated using one‐way ANOVA and the Student's *t* test when data could be assessed as normally distributed. A value of *P* < 0.05 was considered statistically significant.

## 3 RESULTS

### 3.1 hiHeps have poor proliferative capacity in GCM14 medium

**Figure 1:**
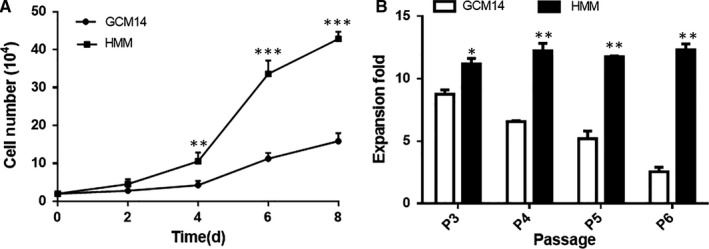
The proliferation and expansion of hiHeps in HMM and GCM14. A, Cell proliferation over 8 d. B, The fold expansion of cells over four generations. All data are presented as means ± SD; n = 3 samples per experimental group. *P < 0.05, **P < 0.01, ***P < 0.001, compared with control (GCM14)

Previously, a chemically defined SFM‐GCM14 was generated using statistical experimental design. First, the proliferation capacity of hiHeps in GCM14 was investigated. The results showed that compared with hiHeps cultured in HMM medium, hiHeps cultured in GCM14 have low proliferative capacity within 8 days (Figure 1A), and the fold expansion was gradually reduced (Figure 1B). As a result of the limited proliferation and expansion in GCM14, it was difficult to support long‐term culture and expansion of hiHeps in vitro*.*

### 3.2 Effect of insulin on hiHep proliferation and gene expression

**Figure 2:**
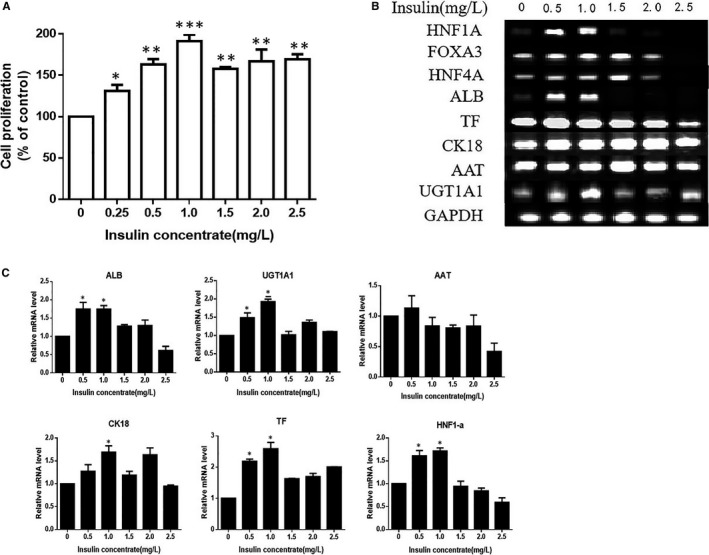
Effect of insulin on the proliferation and relative gene expression of hiHeps in GCM14. A, Proliferation of hiHeps cultured in GCM14 without or with insulin (0‐2.5 mg/L) and measured by CCK‐8 assays. Values are adjusted relative to the non‐treated control, which was set to 100%. B, Semiquantitative RT‐PCR was performed to measure the level of gene expression in hiHeps. C, qRT‐PCR was performed to quantitatively detect the relative gene expression. All data are presented as means ± SD; n = 3 samples per experimental group. *P < 0.05, **P < 0.01, ***P < 0.001, compared with control（0 mg/L insulin）

As a result of the poor proliferative capacity of hiHeps in GCM14 medium, a strategy of using exogenous growth factors to promote cell proliferation was adopted. Studies have revealed that insulin can promote the proliferation and function of several cell types.^22^, ^23^, ^24^, ^25^ In this study, hiHeps were exposed to various concentrations of insulin, ranging from 0 to 2.5 mg/L, and their proliferative activity and expression of important genes were measured in GCM14 at day 4. The results indicated that insulin‐stimulated cell proliferation in a dose‐dependent manner at concentrations up to 1.0 mg/L. Nevertheless, at a concentration of 1.5 mg/L or higher, the proliferation slightly decreased (Figure 2A). Furthermore, the expression of three transcription factors and hepatocyte function‐associated genes were measured using semi‐qRT‐PCR and fluorescence qRT‐PCR. Analysis of the brightness of the electrophoresis band showed that insulin had no effect or a slight effect on the gene expression of *TF*,* CK18* and *AAT* as the concentration increased. However, the gene expression of *HNF1A*, *ALB* and *UGT1A1* significantly increased at insulin concentrations up to 0.5 or 1.0 mg/L (Figure 2B). Further qRT‐PCR analysis showed, consistent with the trend of the results obtained from electrophoresis, the mRNA expression levels of *HNF1A*, *ALB* and *UGT1A1 *increased upon treatment with 0.5 or 1.0 mg/L insulin, most noticeably with 1.0 mg/L (Figure 2C). Taken together, the results indicated that 1.0 mg/L insulin is the optimal concentration to affect hiHep proliferation and liver function gene expression. Hereafter, the SFM with 1.0 mg/L insulin is referred to as GCM14I.

### 3.3 Insulin enhances hiHep proliferation through the Akt1‐Cyclin D1 mediated G1‐S phase progression

**Figure 3:**
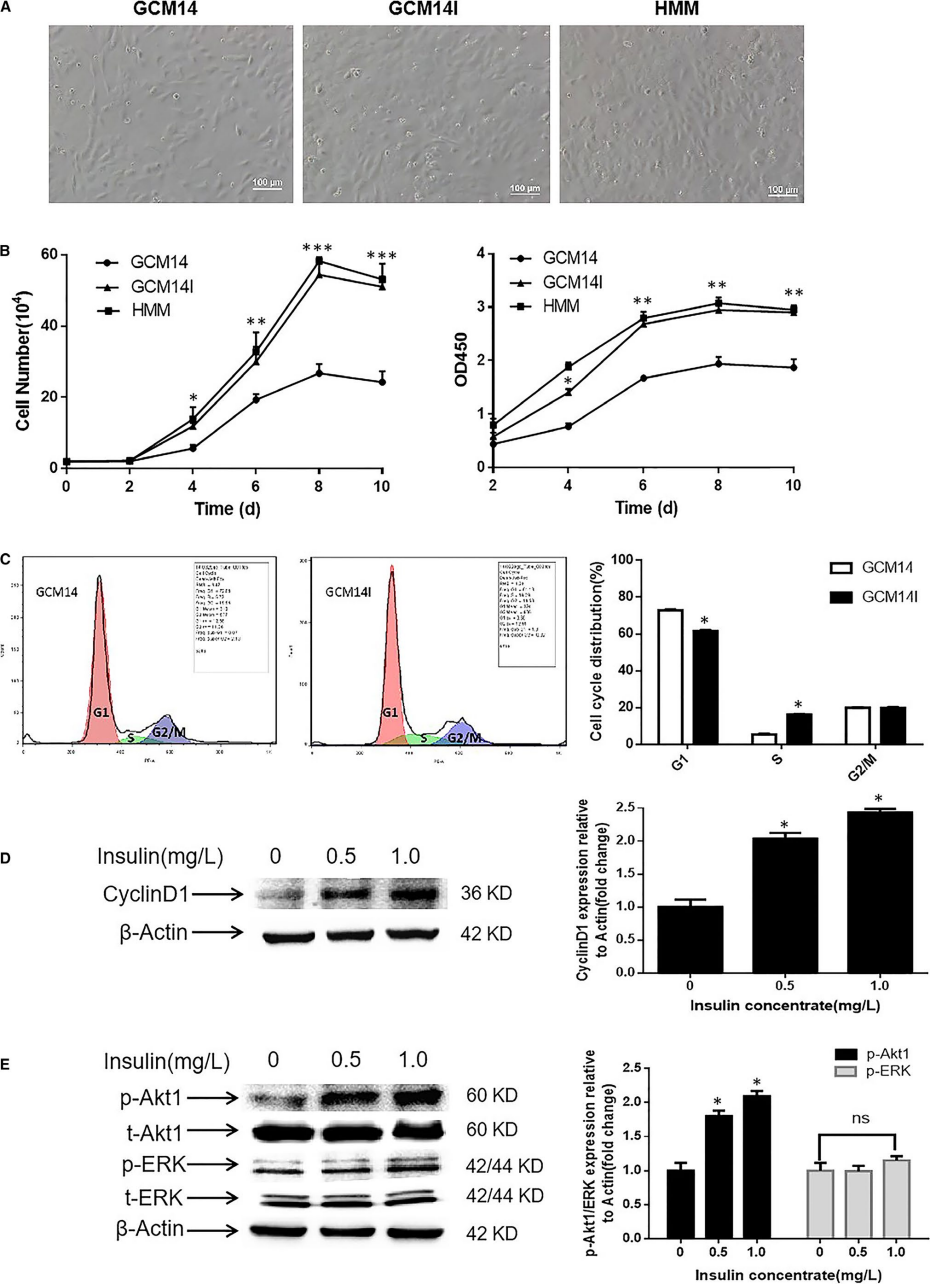
Effect of insulin on cell morphology, growth, viability, cell cycle distribution and the expression of related regulators in hiHeps. A, Photomicrograph of hiHeps cultured in GCM14 without or with 1.0 mg/L insulin and in HMM for 4 d (scale bar, 100 μm). B, Effect of insulin on the growth and vitality of hiHeps in various culture media over 10 d. C, hiHeps incubated in GCM14 with 0 and 1.0 mg/L insulin for 4 d were subjected to flow cytometry, and the percentages of cell cycle distribution (G1, S and G2/M) were measured. D, The expression level of cyclin D1 was determined by Western blotting (actin served as an internal control for protein loading). The bar graph shows the cyclin D1/β‐actin ratio and is expressed as the fold change compared with the control. E, The expression levels of phosphorylated Akt1 and ERK in hiHeps treated with 0, 0.5 and 1.0 mg/L insulin. All data are presented as means ± SD; n = 3 samples per experimental group. *P < 0.05, **P < 0.01, ***P < 0.001

As 1.0 mg/L insulin significantly promoted the proliferation of hiHeps, we next investigated the morphology, growth and viability of hiHeps in GCM14I. First, the morphology of hiHeps at day 4 was evaluated. The typical epithelial‐like polygonal morphology was not altered when hiHeps were treated with insulin (Figure 3A). Then, the growth curves and viability of hiHeps were determined in the presence of insulin. The results suggested that insulin significantly promotes hiHep proliferation and viability and is as effective as HMM. (Figure 3B). As the proliferation of cells is closely related to cell cycle progression, we further investigated the effect of insulin on cell cycle distribution.^26^, ^27^ hiHeps were treated with insulin (0 and 1.0 mg/L) for 4 days and were then tested by flow cytometry. The results showed that adding 1.0 mg/L insulin decreased the percentage of cells in G1 phase, while increasing the percentage of cells in S phase, demonstrating that the G1‐S cell cycle transition was accelerated (Figure 3C). Numerous studies have shown that cyclin D1 plays an important role in regulating cell proliferation and is the key to cell cycle progression at G1‐S phase.^28^, ^29^, ^30^ Thus, the protein expression level of cyclin D1 in hiHeps treated with or without insulin was determined. The results showed that insulin significantly increased the protein expression of cyclin D1 compared to that of the control (Figure 3D), which showed that cyclin D1 was involved in insulin‐induced hiHep proliferation. In general, the effect of insulin on the promotion of cell proliferation is mainly modulated by protein kinase B1 (Akt1) or extracellular regulated protein kinase (ERK).^31^, ^32^ Therefore, the effect of insulin on the phosphorylation of Akt1 and ERK in hiHeps was investigated. Insulin treatment significantly increased the level of Akt1 phosphorylation, but the phosphorylation levels of ERK remained unchanged (Figure 3E). Together, these findings suggest that insulin promotes the proliferation and viability of hiHeps in GCM14 as effectively as HMM, and this effect occurs as a result of the cyclin D1‐mediated G1‐S phase progression, which involves increased Akt1 activity.

### 3.4 Insulin promotes hiHep proliferation via augmented cyclin D1 expression that is mediated by the Akt1/mTOR/p70S6K and Akt1/p53 pathways

**Figure 4:**
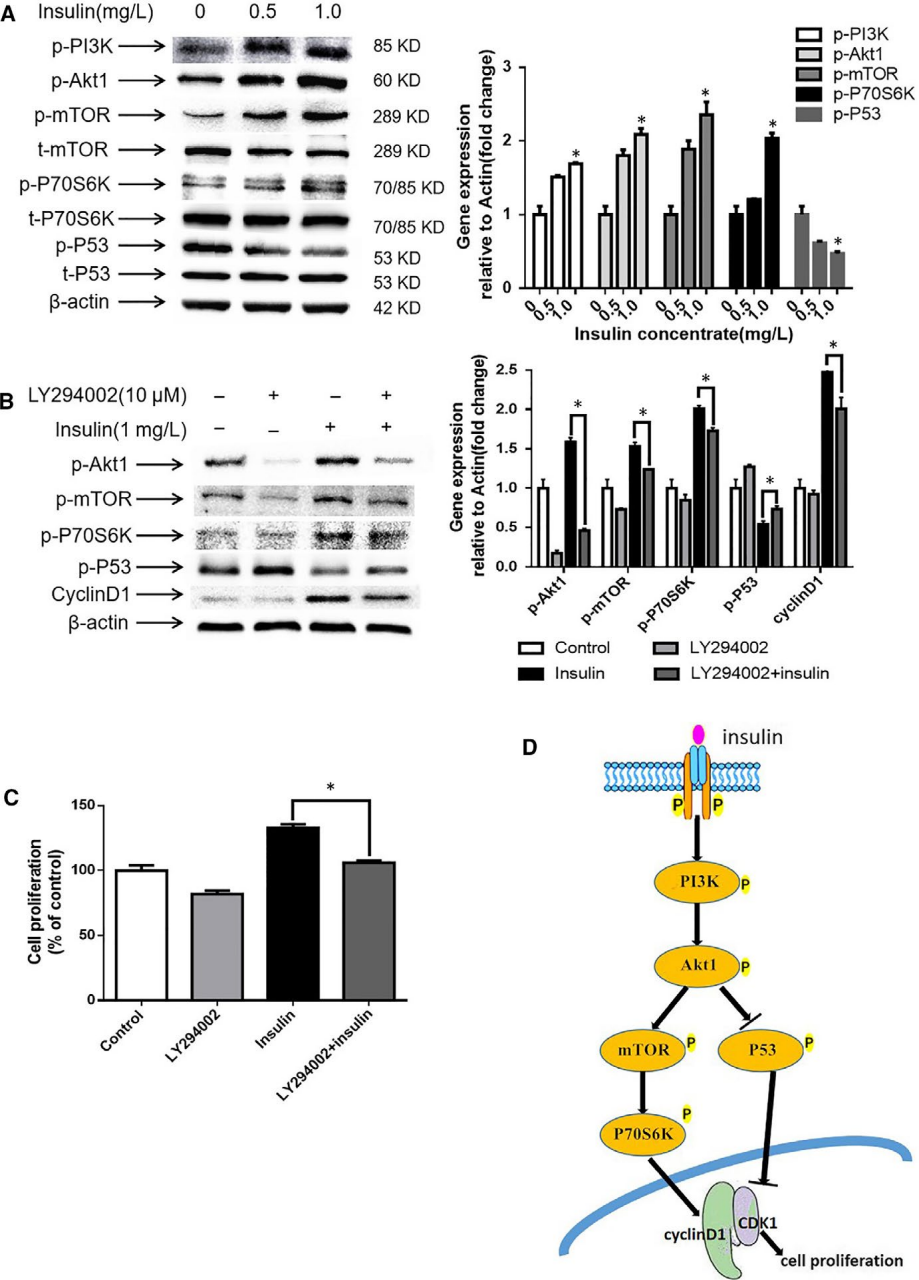
Treatment with insulin stimulates theAkt1/mTOR/p70S6K and Akt1/P53 pathways, and LY294002 treatment decreased the stimulation of insulin at the protein level of factors involved in these signalling pathways. A, Western blot analysis results showing the protein expression of members of the Akt1 pathway after insulin treatment. B, Western blot analysis results showing the protein expression after treatment with LY294002. C, Akt1 inhibition attenuated the pro‐proliferation effect of insulin. D, Signalling pathway of insulin promoted hiHep proliferation. All data are presented as means ± SD; n = 3 samples per experimental group. *P < 0.05

To further investigate the possible molecular mechanisms responsible for insulin‐induced proliferation in hiHeps, we next detected the expression of relevant proteins involved in the AKT signalling pathway in cells treated with different concentrations of insulin (0, 0.5 and 1.0 mg/L). AKT is a key effector protein downstream of PI3K, and activation of PI3K kinase leads to phosphorylation of AKT;^33^ these signalling cascades phosphorylate mTOR, p70S6K or down‐regulate the expression of P53, which act as transcription factors that influence the cell cycle and regulate cell proliferation.^14^, ^34^ Therefore, the protein levels of p‐PI3K, p‐Akt1, p‐mTOR, p‐p70S6K and p‐P53 were evaluated. The results showed that insulin significantly increased the protein expression levels of p‐PI3K, p‐Akt1, p‐mTOR and p‐p70S6K, while the p‐P53 levels were markedly reduced in a dose‐dependent manner compared with those of the control group (Figure 4A). To verify the role of the signalling pathway, hiHeps were treated with 1.0 mg/L insulin in the presence or absence of an AKT inhibitor LY294002 (10 μmol/L), and the effects of insulin on protein expression and cell proliferation were measured. The results showed that LY294002 significantly attenuated the insulin‐induced increases in p‐Akt1, p‐mTOR, p‐p70S6K and cyclin D1 expression levels and increased the expression of p‐P53 (Figure 4B). Additionally, LY294002 also reduced insulin‐stimulated hiHep proliferation (Figure 4C). Together, these results suggest that insulin regulates the proliferation of hiHeps through two different mechanisms by increasing the expression of cyclin D1 via the Akt1/mTOR/p70S6K signalling pathway, and by down‐regulating the expression of the P53 protein, which inhibits cyclin D1 activity (Figure 4D). Both of these mechanisms drive cell cycle progression and promote hiHep proliferation.

### 3.5 Effect of insulin on the liver function of hiHeps

**Figure 5:**
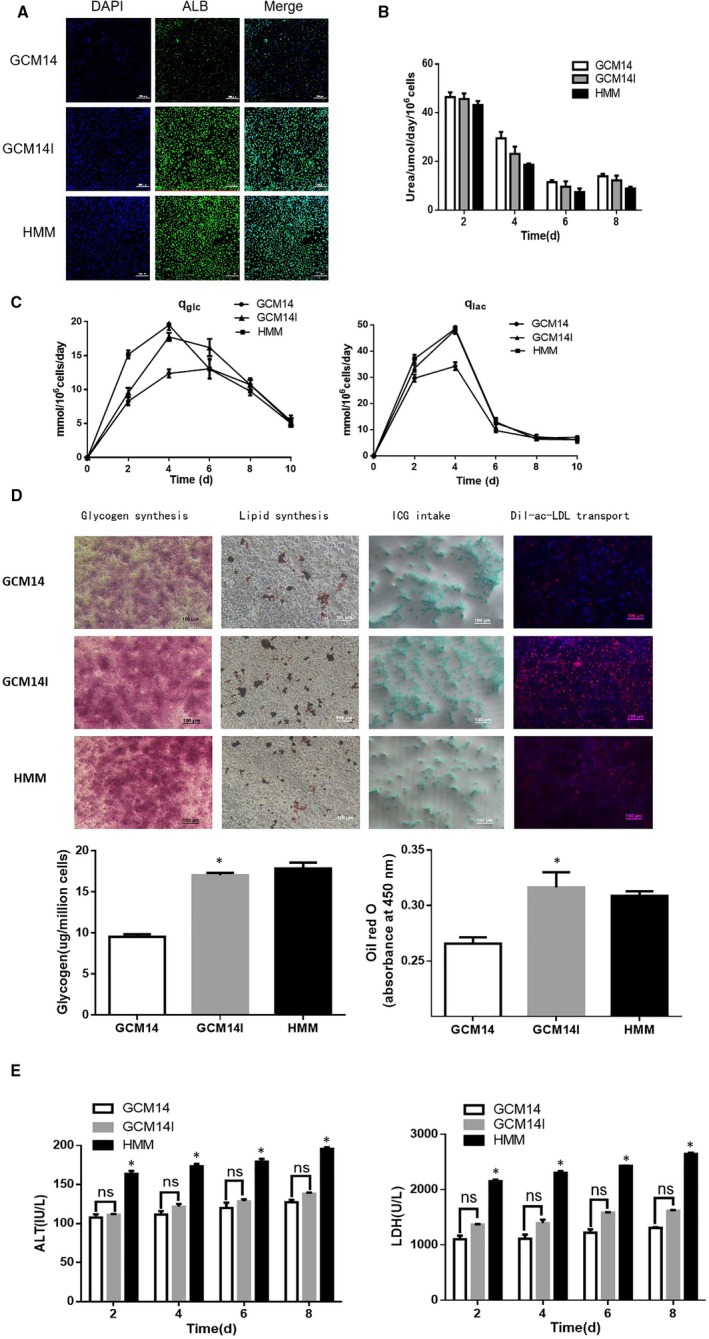
Effect of insulin on the liver function of transdifferentiated hiHeps in GCM14. A, The synthesis of ALB in hiHeps by immunofluorescence staining when cultured in GCM14, GCM14I and HMM (green). B, Urea synthesis. C, Glucose consumption and lactate production. D, Glycogen synthesis, lipid synthesis, ICG intake and Dil‐ac‐LDL transport. E, Activity of ALT and LDH secreted into the supernatant. All data are presented as means ± SD; n = 3 samples per experimental group. *P < 0.05

Previous results have indicated that the addition of 1.0 mg/L insulin to GCM14 significantly promotes hiHep proliferation. However, the harvested cells are also required to have normal liver function. Thus, the effect of insulin on hiHep function was investigated. Albumin and urea are synthesized in the liver, which are important markers that reflect the detoxification function of the liver.^35^ The results showed that the synthesis of ALB was increased in hiHeps after the addition of insulin (Figure 5A). While the secretion of urea did not increase, but hiHeps had increased urea secretion capacity in SFM, compared with HMM (Figure 5B). Next, we found that the consumption of glucose and the production of lactic acid were both increased in GCM14I as effectively as in HMM (Figure 5C). Furthermore, the staining and quantitative analysis of glycogen and lipids showed that insulin promotes the synthesis of glycogen and lipids in cells, and hiHeps have a better ability to absorb ICG and transport DiI‐labelled ac‐LDL in GCM14I than in GCM14 (Figure 5D). Generally, ALT and LDH can be used as sensitive indicators of liver function decline.^36^ Analysis of ALT and LDH in culture supernatant revealed no noticeable difference after insulin treatment, but the release of ALT and LDH were lower in SFM, which indicates that hiHeps undergo a low level of cell damage when cultured in SFM (Figure 5E). These results suggest that insulin promotes hiHep albumin synthesis, glucose consumption, glycogen synthesis and lipid accumulation. Compared with HMM, hiHeps have increased urea secretion capacity, hepatocyte ICG uptake capacity, ac‐LDL transport capacity and undergo low levels of cell damage in GCM14I.

### 3.6 Insulin increases the expression of ALB and UGT1A1 through HNF1A

**Figure 6:**
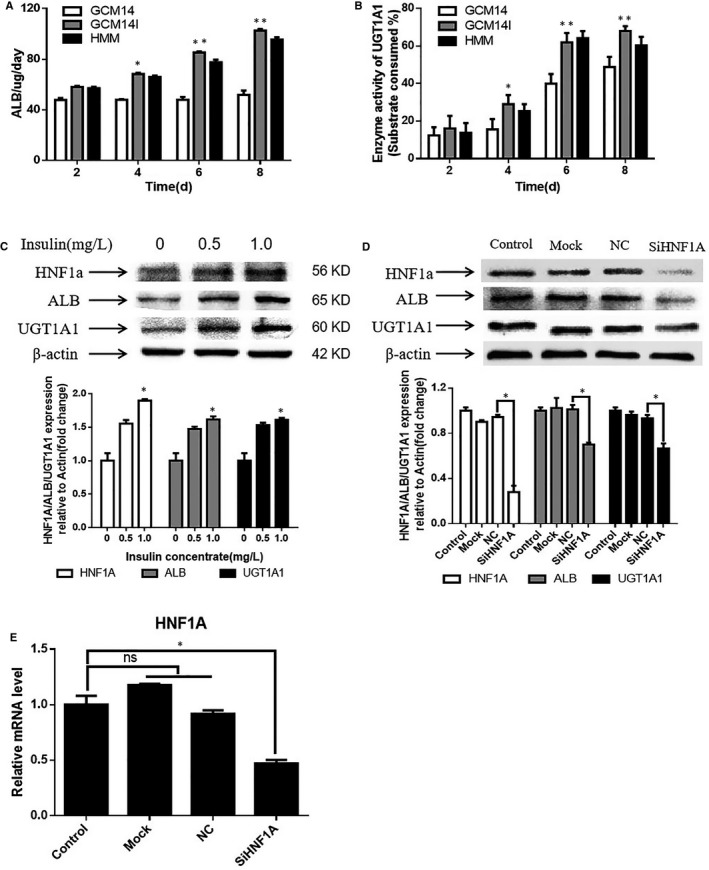
The effect of insulin on the expression of ALB and UGT1A1. A, Secretion of albumin in cell culture supernatant. B, Enzyme activity of UGT1A1 in hiHeps. C, The protein expression of ALB, UGT1A1 and HNF1A in hiHeps treated with 0, 0.5 and 1.0 mg/L insulin by Western blotting. D, Expression of the abovementioned proteins was measured in hiHeps transfected with siHNF1A. E, qRT‐PCR was performed to analyse the level of related genes in hiHeps after transfection. All data are presented as means ± SD; n = 3 samples per experimental group. *P < 0.05, **P < 0.01

ALB is an important plasma protein with antioxidant and detoxification abilities,^37^ and UGT1A1 is a critical phase II metabolic enzyme responsible for the detoxification of bilirubin,^38^ both of these proteins are key markers that are used for assessing hepatocyte function. Previous results showed that the gene expression of *ALB*, *UGT1A1* and *HNF1A* were significantly increased after treatment with 1.0 mg/L insulin (Figure 2B,C). Thus, we measured the secretion level of ALB in hiHeps by ELISA and the enzyme activity of UGT1A1 by substrate consumption, with or without insulin treatment. The results showed that insulin treatment increased the expression level of ALB and the enzyme activity of UGT1A1 during cell culture (Figure 6A,B). Western blot analyses also showed that insulin increased the protein expression of ALB and UGT1A1 in hiHeps, as well as the expression of HNF1A (Figure 6C). As HNF1A is an exogenously induced transcription factor, we have been suggested that insulin may increase the protein expression of ALB and UGT1A1 by affecting HNF1A. To verify this, siHNF1A was transfected into hiHeps that were treated with insulin. After transfection with siRNA, the protein level of HNF1A in hiHeps was significantly decreased compared with that of the control, and the protein expression of ALB and UGT1A1 was also reduced (Figure 6D). The qRT‐PCR results showed that gene expression of *HNF1A* was reduced after transfection, which was consistent with the Western blotting results (Figure 6E). Based on these results, we propose that insulin increases the expression of ALB and UGT1A1 via HNF1A.

### 3.7 Insulin treatment supports hiHep proliferation and stable expression of specific genes during long‐term passaging

**Figure 7:**
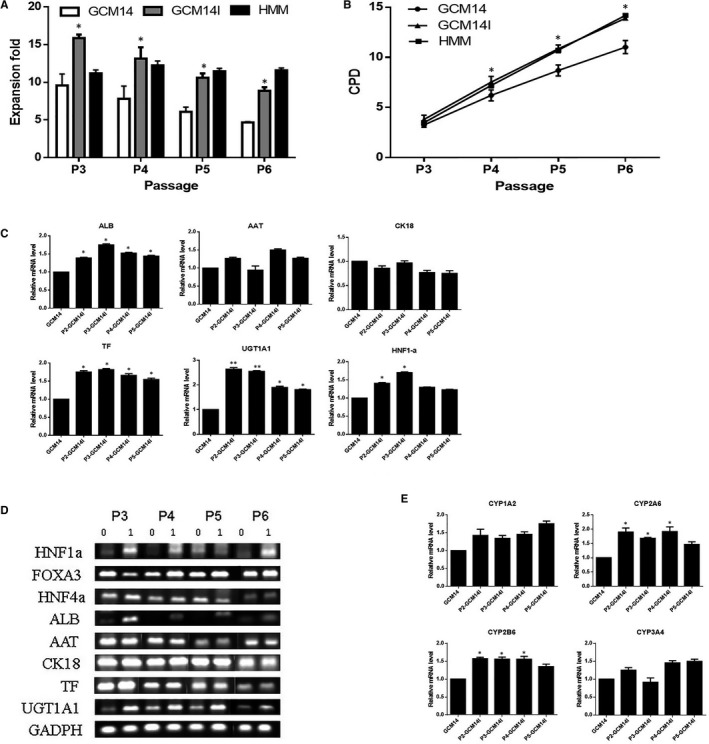
The effect of insulin on hiHep proliferation and expression of specific genes during long‐term passaging in GCM14I. A, The effect of insulin on hiHep expansion (fold increase) during long‐term passage in different culture media and B, cumulative population doubling (CPD). C, Hepatocyte‐specific gene expression by semi‐qRT‐PCR and qRT‐PCR analysis. D, Expression of the CYP gene in transdifferentiated hiHeps with or without insulin in GCM14. All data are presented as means ± SD; n = 3 samples per experimental group. *P < 0.05

hiHeps are promising seeding cells for BALs for hepatic disease treatment. These results indicated that 1.0 mg/L insulin promotes hiHep proliferation and specific, functional gene expression in SFM as effectively as in HMM. As a sufficient number of functional hiHeps are required for clinical treatment using a BAL, the effect of insulin on the long‐term passage of hiHeps was investigated. The addition of insulin to GCM14 remarkably increased cell proliferation, and this effect was maintained for at least four generations (Figure 7A). After four generations, the cumulative population doubling (CPD) was approximately 15 in GCM14I, which was equal to that of HMM (Figure 7B). PCR analysis revealed that the expression of three transcription factors and hepatocyte function‐associated genes was stable during long‐term passaging after adding 1.0 mg/L insulin (Figure 7C,D). Further, the expression of the phase I metabolic enzyme CYP genes was tested by real‐time qRT‐PCR, and the results showed that the level of the CYP genes can be stably maintained during long‐term passaging. (Figure 7E). Together, these results suggest that insulin enables hiHep proliferation and stable expression of specific genes during long‐term passaging. Additionally, the number of functional hiHeps harvested was sufficient to meet clinical requirements for a BAL when cultured in GCM14I medium.

## 4 DISCUSSION

Previous studies have demonstrated that insulin plays an important role in promoting the proliferation of various cell types.^22^, ^23^, ^24^, ^25^ However, whether insulin regulates the proliferation and function of human hepatocytes, especially transdifferentiated cells, remains poorly understood. Previously, we first screened a serum‐free media through Plackett‐Burman design,^11^ and then obtained a medium that can maintain the growth and function of hiHeps through initial optimization, named GCM14 medium. In this report, our results demonstrated that insulin has an obvious effect on hiHep proliferation and specific liver functions at a concentration of 1.0 mg/L in GCM14 medium. hiHeps were transdifferentiated from human fibroblasts. Several studies have reported that 10‐100 mg/L insulin significantly increased the proliferation of human fibroblasts.^39^, ^40^ However, for the cells obtained by transdifferentiation, our results showed that insulin at 0.5‐1.0 mg/L had a significant effect on hiHep proliferation. This was possibly related to the expression of insulin receptors on the cell surface.

In general, the effect of insulin on promoting cell proliferation mainly occurs through the AKT and ERK pathways, and cell proliferation is closely related to the cell cycle. hiHeps were transdifferentiated from fibroblasts, and research has demonstrated that in fibroblasts, insulin‐induced ERK activation is necessary for cell proliferation.^41^ However, our study showed that insulin increased cyclin D1 and p‐Akt1 expression in hiHeps, which was different from that in fibroblasts. Studies have shown that p70S6K or P53 could act as transcription factors that regulate cell proliferation by affecting the expression of cyclin D1.^42^, ^43^ For example, previous studies showed that insulin stimulates goose liver cell growth by activating the PI3K/AKT/mTOR/p70S6K signalling pathway,^14^ and there are also studies indicating that activation of P53 induced liver damage and suppressed the rate of liver cell proliferation.^44^ Although there are many studies on the effects of insulin on cell proliferation, the effect of insulin on human hepatocytes, especially on transdifferentiated hepatocytes, has been explored little. Our results suggest that insulin regulates the proliferation of hiHeps through two different mechanisms by increasing the expression of the cell cycle marker cyclin D1 via the Akt1/mTOR/p70S6K pathway and by down‐regulating the expression of the P53 protein. Both of these mechanisms drive the cell cycle and promote the proliferation of hiHeps.

hiHeps are promising seeding cells for BALs for liver failure treatment. Previously, we showed that 1.0 mg/L insulin significantly promoted hiHep proliferation. However, in addition to proliferation, hiHeps must also have normal liver function after culture in GCM14I. Insulin has extensive biological effects that regulate cell metabolic function. For example, research has showed that insulin is an important regulator of liver‐specific cytochrome P450 gene expression.^16^ Other studies have also shown that insulin can promote lipid synthesis, protein synthesis and glycogen synthesis.^13^, ^15^ However, the effect of insulin on the function of human hepatocytes, especially on transdifferentiated cells in SFM has been rarely reported at present. Our study revealed that transdifferentiated hiHeps have the ability to synthesize and secrete albumin and that insulin promotes hiHep albumin synthesis, glucose consumption, glycogen synthesis and lipid accumulation. Additionally, hiHeps have increased urea secretion capacity, transport capacity and undergo low levels of cell damage in GCM14I compared with those of HMM. These results showed that insulin also affects transdifferentiated cells. However, it is still unclear whether the mechanism through which insulin affects the function of transdifferentiated cells is similar to that of normal cells.

In this study, we found that the mRNA expression levels of* ALB, UGT1A1 *and the transcription factor *HNF1A* were increased after 1.0 mg/L insulin treatment. Further, the secretion of ALB in hiHeps was detected by ELISA, and the enzyme activity of UGT1A1 was detected by substrate consumption. These results also showed that insulin increased the expression of ALB and UGT1A1. Research has shown that the expression level of *HNF1A* is positively correlated with the expression of *ALB* and *UGT1A1* at the mRNA and protein level,^45^ and that there are binding sites of *HNF1A* in the promoter region of the *ALB *and *UGT1A1* genes.^46^ Therefore, we have been suggested that insulin may increase the protein expression of ALB and UGT1A1 by affecting the level of *HNF1A*. We performed the protein level analysis by Western blot analyses and verified the results by down‐regulating the expression of *HNF1A* by siRNA transfection. The results showed that insulin affected the expression of ALB and UGT1A1 via HNF1A. However, we do not know how insulin regulates the expression of HNF1A, and the literature reports very little on this topic. Finally, the results demonstrated that insulin treatment supports hiHep proliferation and stable expression of specific genes during long‐term passaging in SFM. Although it is theoretically possible to generate 10^10 ^functional hiHeps in vitro in GCM14I, when actually culturing these cells, not only is the cost high but it is also difficult to obtain a high enough cell number for a BAL in a short time. Therefore, we need to achieve high‐density culture of hiHeps in serum‐free GCM14I, including microcarrier culture and three‐dimensional perfusion culture, which will create a solid basis for the clinical application of hiHep‐BALs.

## CONFLICT OF INTEREST

The authors have declared that no competing interest exists.

## AUTHORS’ CONTRIBUTIONS

Ce Gu performed all the experiments and wrote the manuscript. Panpan Li and Wei Liu participated in preparation of SFM. Yan Zhou and Wen‐Song Tan contributed to the conception, design of the work or of parts of it, and its interpretation.
